# “*You’re just in such crisis mode…frantic to get through the next day”*: an interpretative phenomenological analysis of adjustment experiences among carers of patients with advanced oesophago-gastric cancer

**DOI:** 10.1186/s12904-025-01810-6

**Published:** 2025-07-01

**Authors:** Cara Ghiglieri, Martin Dempster, Lisa Graham-Wisener

**Affiliations:** Centre for Improving Health-Related Quality of Life, School of Psychology, Queen’s University Belfast, Belfast, BT7 1NN UK

**Keywords:** Informal carers, Adjustment, Coping, Oesophago-gastric cancer, Upper GI cancer, Qualitative, Palliative care

## Abstract

**Background:**

In advanced oesophago-gastric cancer, rapid disease progression and complex symptoms contribute to a distinct caring context. Yet little is known about how those providing informal care interpret and adjust to these experiences, despite increasing recognition that this plays a key role in shaping psychological well-being. This study aimed to comprehensively explore these carers’ adjustment experiences, identifying opportunities for improved care and support.

**Methods:**

Semi-structured interviews were conducted with ten purposefully recruited individuals who had cared for someone with advanced oesophago-gastric cancer in the UK. The interviews were audio-recorded, transcribed verbatim, and analysed using interpretative phenomenological analysis.

**Results:**

Carers experienced a demanding adjustment process as they took on complex nutritional and emotional responsibilities, often with limited guidance or support. The rapid trajectory and disruption to roles and routines left many feeling overwhelmed and unable to meet their own needs. Ongoing efforts to seek understanding, preserve connection, and focus on the present supported their attempts to manage uncertainty and sustain meaning.

**Conclusions:**

The findings reflect the unique complexities of advanced oesophago-gastric cancer care, pointing to the need for support models that acknowledge the complex, relational, and often hidden aspects of adjustment to caring in this context.

**Supplementary Information:**

The online version contains supplementary material available at 10.1186/s12904-025-01810-6.

## Background

Oesophago-gastric cancer includes cancers of the oesophagus, stomach, or oesophago-gastric junction. This aggressive and complex disease affects approximately 15,000 people each year in the UK [1, 2] and around 1.5 million globally [3]. It presents unique challenges in diagnosis and treatment, largely due to non-specific symptoms and the difficulty of early detection [4, 5]. As a result, most cases are diagnosed at an advanced stage [6], progress rapidly [7], and are associated with poor prognoses [8]. Five-year survival rates remain low, at 17% for oesophageal cancer and 21.5% for gastric cancer [8, 9], placing them among the six least survivable cancers worldwide [10].

Providing informal care for someone with advanced disease has been described as a *‘‘fraught experience that extends well beyond the death of the person receiving care”* [11]. Informal care refers to unpaid support typically offered by family members, friends, or close others, and may involve a broad range of responsibilities including personal care, emotional support, and coordinating medical appointments [12–15]. While many take on this role willingly [16], the ongoing and multifaceted nature of caring can result in cumulative strain. This strain is commonly conceptualised as carer burden, a multidimensional construct that captures the physical, emotional, social, and financial impacts of providing care [17]. Higher levels of burden have been consistently associated with poorer psychosocial outcomes, including anxiety, depression, and reduced quality of life [18].

Whilst it has been recognised that patients with advanced oesophago-gastric cancer rely extensively on support from those close to them [19, 20], our recent systematic review revealed no studies have explored the perspective of the person providing this care [21]. Some evidence does exist from curative contexts. For instance, informal carers supporting people undergoing treatment for oesophago-gastric cancer have reported increased mental strain, psychological distress and lower wellbeing [22–24]. While these findings offer some insight into the emotional demands of care, they reflect experiences within curative treatment contexts, where prognosis is generally more favourable and care demands may differ. Broader research across cancer populations have shown that the burden on carers of people with cancer intensifies as the disease progresses [25]. Beyond the physical and emotional difficulties associated with providing care, these individuals must also come to terms with the prospect of losing their loved one [26–28].

In the context of advanced oesophago-gastric cancer, several factors would likely exacerbate the challenges faced by informal carers. The disease is typically characterised by high symptom burden and rapid functional decline [29], alongside a prolonged treatment pathway that involves multiple investigations, transitions between services, and coordination across medical specialties [30–34]. Common symptoms such as dysphagia, pain, fatigue, weight loss, and bowel changes often intensify as the disease progresses, significantly diminishing quality of life and increasing care needs [29, 33]. Individuals receiving palliative treatment for advanced oesophago-gastric cancer frequently report higher levels of psychological distress and poorer overall wellbeing compared to those undergoing curative treatment [29, 35]. Qualitative studies with patients describe a ‘complex life situation’ characterised by existential anxiety [36], loss of dignity [37], and disruptions to social and relational roles [20]. Although these studies centre the patient perspective, they highlight that informal carers are closely involved throughout the illness trajectory and often share in these experiences [19, 20]. Acknowledging this is important, as emerging evidence suggests that patient and carer psychological wellbeing is interdependent, shaped not only by individual experiences but also by each other’s perceptions and emotional states [38–40]. This has been seen in curative oesophageal cancer, where carers’ illness perceptions were found to influence the patients’ levels of psychological distress [41].

Yet while many informal carers experience psychological strain, others report maintaining wellbeing or even experiencing personal growth through the caring role [42–44]. To better account for this variability, research has increasingly turned to the concept of adjustment. Existing research with carers has defined adjustment to encompass the psychophysiological outcomes of coping [42]. However, more recent theoretical perspectives conceptualise adjustment as a dynamic and interpretive process through which individuals make sense of their experiences, respond to shifting demands, and adapt over time [45–47]. Understanding adjustment in this process-oriented way can offer critical insights into what shapes positive or negative psychosocial outcomes [47]. These insights can, in turn, inform the development of more responsive, contextually attuned support systems that recognise the evolving needs of carers.

These considerations are especially relevant in advanced oesophago-gastric cancer, where national clinical guidance provides limited recognition of informal carers’ support needs [48]. This likely reflects the broader absence of empirical evidence into the most effective service models to support those affected by oesophago-gastric cancer [49, 50] Individuals who have previously provided informal care and are now bereaved can offer valuable retrospective insights into the experience of caring across the illness trajectory, from diagnosis and treatment through to decline, death, and the bereavement period [51]. Bereavement research is widely accepted as a valid and ethical means of exploring care experiences and is frequently used to evaluate the quality and impact of end-of-life care [52–54].

The study aimed to explore how people who have cared for someone with advanced oesophago-gastric cancer interpret their lived experiences, and how this relates to their process of adjustment.

## Methods

### Design

**Fig. 1:**
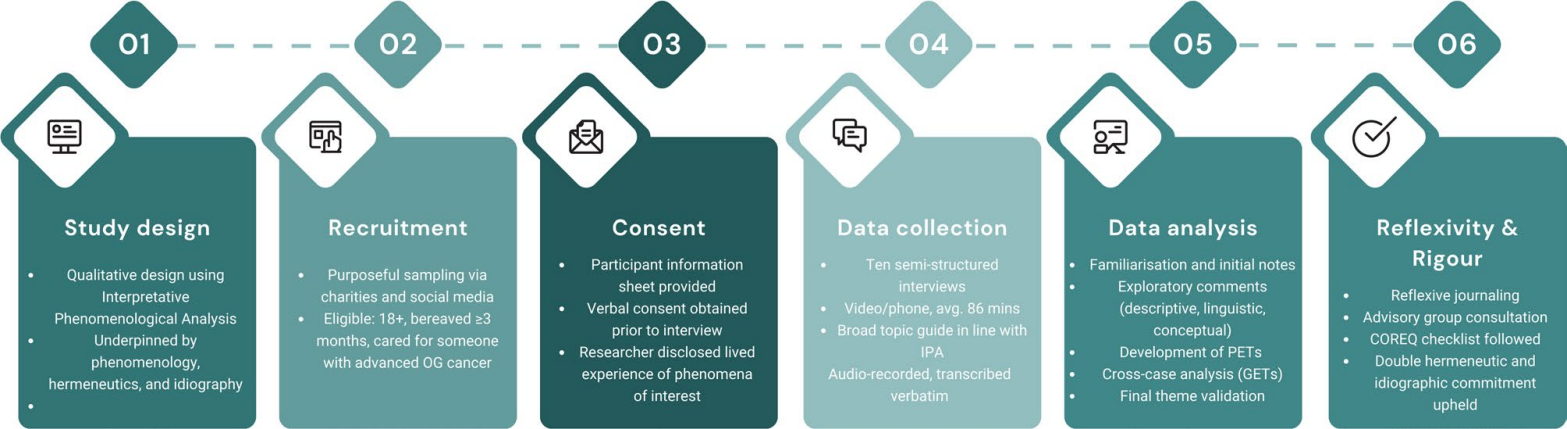
Key stages of the research process

This study adopted a qualitative methodological design informed by Interpretative Phenomenological Analysis (IPA) [55], thus it was underpinned by the philosophies of phenomenology, double hermeneutics and idiography. IPA’s epistemological approach is rooted in interpretative phenomenology [56], hence it is particularly well-suited to understanding how individuals make sense of their lived experiences and the meanings they attribute to those experiences [57]. By employing IPA, we therefore aimed to provide a comprehensive understanding of how informal carers experience (phenomenology) and interpret (hermeneutics) the act of caring for someone with advanced oesophago-gastric cancer. In line with IPA’s idiographic commitment, we conducted detailed case-by-case analyses before examining patterns across participants and exploring points of convergence and divergence. Overall, this design enabled us to identify key targets for care and support, grounded in the participants lived experiences. This aligns with national priorities to provide person-centred support for informal carers as a core component of palliative and end-of-life care in the UK [58]. A visual representation of the methodological steps is presented in Fig. 1.

### Identification and recruitment

Purposeful sampling was used to recruit individuals who: (i) had provided informal care to someone with advanced oesophago-gastric cancer in the UK, (ii) were at least three months since the death of the person they cared for, and (iii) were over 18 years of age. Small, homogeneous samples of ten or fewer are recommended in IPA to support idiographic, in-depth analysis of participants’ accounts [55, 56]. We arrived at ten participants as there was significant interest in the study, and we felt that maximising the recommended sample size would support both the richness of individual accounts and the potential for nuanced cross-case analysis, while remaining consistent with the idiographic focus of IPA [55]. Recruitment took place via UK-based charities, support groups, and social media. Interested individuals contacted the researcher (CG) directly and were sent a participant information sheet. A follow-up call allowed for eligibility screening, discussion of relevance to their experience, and time to ask questions.

### Data collection

Semi-structured interviews were conducted with ten bereaved informal carers between February and March 2021 by the researcher (CG), via video conferencing (*n* = 9) or telephone (*n* = 1). Interviews lasted an average of 86 min. Verbal consent was obtained and recorded prior to each interview. Basic socio-demographic information (e.g. age, relationship to the deceased, time since bereavement) was gathered at the start of each interview, which was attended only by the participant and researcher. The female researcher (CG) conducted this study as part of her PhD and had received in-depth training in qualitative methods and IPA. She is a committee member of OGCancerNI, a Northern Ireland-based charity supporting people affected by oesophago-gastric cancer. She also has personal experience of caring for her mother during her illness with advanced oesophago-gastric cancer and of bereavement following her mother’s death. Although there was no prior relationship with participants, they were informed of her personal connection to the topic and her motivation for the research. In keeping with IPA’s phenomenological orientation [55, 56], a broad topic guide was developed for this study, including open-ended, non-directive questions and minimal prompts (Supplementary Material 1). The guide was reviewed by a Patient and Carer Advisory Group to ensure relevance and sensitivity. Example questions included: *“Could you tell me about your experience of your close person being diagnosed with oesophago-gastric cancer?”*, with follow-up prompts such as *“What was that like?”* and *“How do you feel about this now?”* This enabled the interview to progress organically through conversation between the researcher and participant, reflecting the aims of phenomenology which orientates to a personal perception or account [59]. Interviews were audio-recorded and transcribed verbatim by the researcher. Only the researcher and the participant were present during the interview. Participants were provided with a debrief sheet and signposting to support resources following the interview.

### Data analysis

The interview transcripts were analysed using IPA [55], following an iterative and inductive cycle. First, the researcher (CG) listened to the audio recording of the interview and read through the transcript several times to facilitate familiarisation and fully immerse herself in the original data. Initial observations and thoughts were freely recorded on a hard copy of the transcript, with no parameters on what could be commented on. The next reading of the transcript involved making exploratory comments from the in-text annotations, noted in the right-hand margin, including descriptive, linguistic and conceptual comments. The researcher paid close attention to key objects of concern (i.e. relationships, processes, events) and the meaning of those things for the participant. From these exploratory notes, experiential statements were identified, in the form of concise statements which align with psychological concepts. These experiential statements were then charted and mapped to determine how they fit together, and a clustered list was formed to create a list of interconnected statements. These were then named to form personal experiential themes (PETs) and consolidated into a table. The second researcher (LGW) checked the interpretation of these themes from the transcript at this point. This process was repeated for all transcripts, paying attention to the individuality of each case. The PETs across transcripts were then assessed, with preference given to themes which appeared prevalent and meaningful. From this, a list of group experiential themes (GETs) was developed (Supplementary Material 2). A final read-through of the transcripts was undertaken to ensure the themes were grounded in the data. The development of personal and group experiential themes was discussed with the second and third researchers (LGW & MD) to increase validity.

### Reflexivity

Shaw (2010) argues that reflexivity is a fundamental aspect of experiential qualitative research in psychology, in which the self and other, and the relationship between them, are explicitly examined as part of the research process [60]. In this study, the researcher (CG) approached the work from an ‘insider’ perspective [61], drawing on her lived experience of caring for her mother during her illness with oesophago-gastric cancer and of bereavement following her death. She experienced significant challenges while caring for her mother, hence from an ontological standpoint, she firmly believes that ‘*there is a real world out there’*, which justifies the need for data collection to improve patient and carer experiences [62]. However, she acknowledges that individuals interpret and experience the same phenomena in diverse ways, which fuelled her interest in exploring how others make sense of their experiences of caring for someone with advanced oesophago-gastric cancer. Throughout the research process, she therefore engaged with a Patient and Carer Advisory Group who helped her to maintain a balanced view. The group members offered suggestions on how to approach interviews and allowed the researcher to reflect on interactions with participants after interviews. The researcher also kept a reflexive journal throughout, detailing observations of the environment, interactions with participants and reflections on the process after each interview. This approach is known to contribute to the trustworthiness of the analysis [62] and supports the double hermeneutic approach to analysis [57], where the researcher interprets participants’ interpretations of their experiences.

### Validity and rigour

The study was reported following consolidated criteria for reporting qualitative research (COREQ) guidelines [63]. A checklist is provided in the supplementary information (Supplementary Material 3). Additionally, the study contains qualities that are required for high-quality IPA research [64]. In this sense, the study was phenomenological in that it sought to understand bereaved carers’ experiential accounts and the meanings they ascribe to their experiences, allowing a compelling narrative to unfold. It engaged in the double hermeneutic, as the researcher (CG) paid close analytic attention to participants’ words, who in turn were trying to make sense of their own experiences. Finally, it was idiographic in its commitment to understanding each participant’s account in detail, before carefully exploring patterns of convergence and divergence across the narratives.

## Results

Ten bereaved oesophago-gastric cancer carers were interviewed (see Table 1 for participant characteristics). Fifteen potential participants initially expressed interest in the study; however, five were excluded: two were within 3 months of their loved one’s death, two did not reside in the UK, and one had a close person diagnosed with an unknown primary cancer, suspected to be oesophageal but not confirmed. The sample’s age ranged between 25 and 64 years, and all participants were ethnically white and female. Eight participants’ close person’s primary diagnosis was at an advanced stage, and two had experienced a recurrence. All but two had received treatment with palliative intent via the NHS, the remaining two did not receive treatment. Seven had been diagnosed with oesophageal cancer, two with oesophago-gastric junction cancer, and one with gastric cancer. To preserve anonymity while maintaining a narrative and idiographic focus, participants were assigned pseudonyms rather than ID numbers. This approach aligns with common practice in IPA and allows for clearer engagement with each participant’s individual story [55].

The analysis and interpretation from interviews with ten bereaved carers of patients with advanced oesophago-gastric cancer identified five group experiential themes: (1) Losing ground, [2] Shifting dynamics (3) Navigating the unknown, (4) Finding solace in connection and (5) Reframing perspective. Throughout the findings, quotations to represent the participant’s voice have been embedded as illustrations. To ensure transparency in the idiographic nature of the analysis, a coding tree of group experiential themes is included below (see Table 2).

**Table 1:** Participant characteristics

Pseudonym	Age	Location	Ethnicity	Tumour location	Relationship to patient	Relationship status	Living with care recipient	Other dependents	Time spent caring (months)	Stage of diagnosis	Recurrence	Work circumstances during caring period
Sarah	45–54	England	White	Oesophagus	Daughter	Married	Yes	Yes	2	IV	no	Employed
Kim	55–64	England	White	Oesophagus	Daughter	Long-term relationship	No	No	4	IV	no	Employed
Rebecca	25–34	England	White	Oesophagus	Daughter	Long-term relationship	No	No	14	IV	yes	Employed
Abigail	55–64	England	White	Oesophago-gastric junction	Spouse	Married	Yes	Yes	29	III	yes	Retired
Jane	45–54	England	White	Oesophago-gastric junction	Daughter	Married	No	Yes	1	IV	no	Employed
Deirdre	45–54	Northern Ireland	White	Stomach	Daughter	Married	No	Yes	6	III	no	Other - carer
Beth	55–64	Northern Ireland	White	Oesophagus	Daughter & Sibling	Long-term relationship	No	Yes	8 (mother), 12 (brother)	IV (mother), Unsure (brother)	no	Employed
Maureen	55–65	Northern Ireland	White	Oesophagus	Daughter	Married	No	Yes	15	Unsure (advanced)	no	Employed
Sophie	45–54	Northern Ireland	White	Oesophagus	Daughter	Married	At times	No	12	Unsure (advanced)	no	Retired
Rachel	45–54	Northern Ireland	White	Oesophagus	Sibling	Single	No	Yes	36	IV	no	Retired

**Table 2:** Coding tree for participant themes

Group experiential theme	Notes / Psychological concepts	Participants contributing to the theme
**Losing ground**	Biographical disruption Crisis theory Stress & coping Caregiver burden Loss of control	Abigail, Beth, Deirdre, Jane, Kim, Maureen, Rachel, Rebecca, Sarah, Sophie
**Shifting dynamics**	Attachment Role theory Family systems	Abigail, Beth, Kim, Maureen, Rachel, Rebecca, Sarah, Sophie
**Navigating the unknown**	Health literacy Death literacy Sense-making Self-efficacy Invalidation	Abigail, Deirdre, Beth, Jane, Kim, Maureen, Rachel, Rebecca, Sarah, Sophie
**Finding solace in connection**	Anticipatory grief Attachment Meaning making Resilience Legacy-building	Abigail, Beth, Deirdre, Jane, Maureen, Rebecca, Rachel, Sarah
**Reframing perspective**	Personal growth Psychological flexibility Post-traumatic growth Resilience Meaning-reconstruction	Abigail, Beth, Deirdre, Kim, Jane, Rachel, Sarah, Sophie

### Group experiential theme 1: losing ground

This theme captures the profound disruption and emotional burden experienced by carers throughout the course of caring for someone with advanced oesophago-gastric cancer. Across narratives, participants described how caring responsibilities became increasingly consuming, often overtaking their capacity to think, feel, or act beyond the immediate demands.

Participants often struggled to articulate the totalising nature of the experience, instead drawing on metaphors to convey its impact. Maureen reflected on how she found herself in, *“crisis mode*,* just frantic to get through the next day*,*”* conveying how care felt like a constant state of alert, with little room to process or pause. Similarly, Rachel described the relentlessness of the role, “*It was very*,* very tough. I don’t just mean emotionally*,* I mean physically and emotionally*,* every sort of emotion you’re going through.”* Abigail likened the experience to a *“whirlwind*,*”* noting how *“your whole life becomes a round of appointments and procedure and that kind of carries you along.”* This sense of being propelled by events, rather than guiding them, was echoed in Sophie and Jane’s imagery of a *“conveyor belt”* and a *“roller coaster speeding out of control*,*”* underscoring a shared feeling of powerlessness as the illness intensified.

For Kim, the effects of this upheaval infiltrated every part of her life. Here, the strain was not only logistical but moral; care became something that had to be done, even when personal limits were stretched.

I was 3 h away, I had a daughter at uni, and the dogs, and you know, it was hard […] it was difficult. I had a life… So, for me to work, and then drive down on a Friday and back on Sunday […] or to come back in one day[.]. But it had to be done.

Others similarly described how the all-consuming demands of caring required them to sideline their own needs:

*“I found there was not much time in between to think of anything else*,* and you know*,* of myself*,* because it was just*,* he was the priority.”* (Abigail).

*“I went to every appointment and tried to think positive*,* but looking back*,* no*,* I was just exhausted*,* but I just did it cause I didn’t want to not do it*,* you know.”* (Sophie).

*“Everything just stopped*,* and I didn’t even realise everything had stopped until a few months after dad had gone.”* (Maureen).

For some, this silencing of personal needs extended beyond the practical into the emotional. Beth captured this disconnection between outward functioning and inner experience:

I cried in bed because of the situation, but then I got up in the morning and got on with it. You don’t I don’t know how you do it, because it’s all consuming, but it’s not. You’re living a life that’s not real. I mean, it’s just you’re in a bubble.

Her imagery of a *“bubble”* conveyed a form of emotional dislocation, a way of psychologically containing distress in order to maintain day-to-day functioning. Rebecca’s reflection mirrored this strategy, *“I think at the time I was just in this sort of in this ‘Keep calm*,* carry on’ stage*,* but I can’t remember. I just kinda did what I had to do*,* kind of thing.”* While adaptive in the short term, she also acknowledged that this suppression of needs prevented her from seeking support: *“I think when you go through something like that you don’t realise just how affected you are. So*,* I didn’t at the time*,* I didn’t seek any support from Macmillan or anything like that*,* not at all*,* and neither did my brother.”*

Like Rebecca, Deirdre described how the intensity of care left little space to seek help, and importantly, that support was not offered unless actively pursued, *“I didn’t seek out specific support really*,* because I didn’t really have time to do any specific support.”* Her sense of marginalisation was deepened by a lack of recognition from professionals: *“I actually feel that we had six months of Mummy*,* from diagnosis to death*,* of not being recognised… we were basically her only support.”* Sarah, too, felt they had been *“left to it*,*”* wondering whether her competence had led others to assume she didn’t need help, *“I wonder if that is because I presented as a capable person […] with some medical knowledge? […] I just got on with it.”*

Where formal support was available, its value was evident. Abigail shared, *“I had a brilliant specialist Nurse who was my lifeline. And I could ring her*,* and she would get an appointment for me or anything like that.”* In the absence of formal support, some participants, like Rebecca, turned to informal sources of connection and validation, *“I lived with a friend who had a terminally ill parent so when I was at the house*,* it was nice to talk to them sort of thing.”*

### Group experiential theme 2: shifting dynamics

This theme captures how carers made sense of shifting relational dynamics as their loved ones’ physical health declined. Difficulties with eating, drinking, and digestion not only signalled deterioration but also destabilised familiar roles and routines, requiring carers to renegotiate how love, care, and connection were expressed within the context of physical vulnerability.

Although her sisters’ bodily changes stemmed from the physical effects of the disease, Rachel gave a vivid account of their emotional toll, *“Oesophageal cancer*,* it just takes your basic ability to eat and drink away from you*,* so if you imagine*,* on the hottest day of your life*,* and you cannot get a drink. Just imagine how horrible that feeling is.”* This wasn’t just about nutrition. For many, the disruption to eating represented a loss of intimacy and relational ritual. Beth reflected on the reversal of roles she witnessed as her mother declined, *“It’s very hard to watch*,* incredibly difficult to watch. Seeing my mum eat baby food*,* it was horrific […] You know*,* for a grown adult*,* it’s so demeaning*,* not only are they ill*,* but then they’re treated like a child.”* Her words pointed not only to the indignity of the experience but to its unsettling impact on how she related to her mother.

For Sophie, too, these changes disrupted previously held ideas about how love and care are expressed, “*It’s human nature to feed everybody and support them. You love somebody by feeding them*,* giving them food*,* and making sure they’re all right. And when you can’t socialise*,* can’t do that*,* it’s very difficult.”*

Rachel also spoke of the emotional toll of witnessing her sister’s loss of pleasure in something once central to her identity:

It was really hard to watch someone like her, who was very vivacious, and the life and soul of the party, you know. She was Mrs Christmas, and she loved her food, loved cooking for people all that kind of stuff. It became, hard, to watch someone who loved their food struggle to enjoy the things that they used to enjoy.

As carers adjusted to these changes, many described how their relationships began to shift. Sophie reflected on the discomfort of taking on responsibilities that felt at odds with her place in the relationship: *“You just don’t think you should be doing certain things for your mother. You shouldn’t have to do that.”* Abigail, too, articulated how her role evolved from partner to clinical carer, *“I remember thinking*,* like*,* I’ve become a nurse and a carer more than a wife. You know*,* it was all very clinical.”* Reflecting the unique challenges that people who care for those with oesophago-gastric cancer often manage, she recalled specific moments that emphasised this shift, *“I was having to squirt water through the feeding tube*,* you know*,* to keep him hydrated.”* These altered dynamics were often felt most acutely in moments of vulnerability, such as helping with bathing:

He used to hate, you know, towards the end he needed me to help him get into the bath. He just didn’t have the strength to get himself in and out of the bath and he hated that. You know, he used to say, look at the state of me, let me get some clothes on quickly, you know. He just hated what it was doing to him.

Yet within these altered roles and strained dynamics, participants often maintained a strong sense of relational commitment. Rachel’s reflection captured the weight of what she had witnessed, but also her refusal to distance herself, *“Seeing her like that*,* it was just devastating*,* but I wouldn’t have had it any other way*,* you know.”*

### Group experiential theme 3: navigating the unknown

This theme conveys how a diagnosis of advanced oesophago-gastric cancer thrust many carers into unfamiliar territory, where a lack of knowledge about the illness, its management, and the uncertain journey ahead appeared to exacerbate their struggles.

Early symptoms were often vague or easily misattributed, contributing to delays in help-seeking and, in several cases, a diagnosis that felt abrupt and unanticipated. The limited public awareness of the disease seemed to compound this shock, and several carers reflected with frustration or regret on how signs had been missed or downplayed, both by themselves and within healthcare settings. Sarah questioned this neglect directly, *“I don’t understand why oesophageal cancer is not on people’s radar.”* Similarly, Kim expressed frustration at the lack of public awareness:

I think the key thing is there has to be more awareness out there. There has to be some kind of campaign, people need to be aware that if you’re on Rennie’s or Zantac, or something, by that point, that needs to be an alarm bell for you. Its fine popping a few Rennie’s, but when you get to needing them every day, then it’s a problem.

For many of the carers, uncertainty extended beyond awareness of the illness. It included not knowing how to support their relative, how the illness might progress, or how to respond when formal systems, and their own personal resources were not enough. Some, like Maureen, described a lack of coordination across services, which made navigating support especially difficult:

*Everyone seems to be operating in their own bubble […]*. There were so many people involved. You were going “do I ring the district nurse.” “Do I ring the GP” “do I ring the pharmacist at the hospital?

Kim similarly recalled her struggle to get clear information, *“Trying to get answers from the consultant was very hard […] I think at that point*,* people want answers and to know how long they have got left. But we couldn’t get the answer.”* Sophie’s experience echoed this absence of communication, *“Nobody told her how she would feel. Nobody actually spoke*,* to say*,* ‘this is how it’s going to be*,* to go in your body.’ You need somebody to tell you the truth*,* not just the medical bits”.*

Jane’s account conveyed how this uncertainty and poor communication could amplify emotional distress, *“I didn’t feel like I was doing a good enough job […] it was really difficult*,* and we weren’t sure if Mum was in pain because she would groan every time.”* She also described a moment of regret when limited information about sedation left her unprepared and unable to say goodbye to her mother:

She just screamed and screamed and screamed. And it was just, she was so distressed, and she didn’t know who any of us were […] So they just didn’t tell me what would happen when she got completely sedated. So, we didn’t get to say goodbye.

In the face of ambiguity, active information-seeking offered as a way to make sense of their circumstances. Beth described how this pursuit of knowledge almost became a new identity, *“I had never heard of the oesophageal cancer. Never had heard of it and I mean*,* now I’m like the oracle on it.”* Her efforts to understand the illness also influenced those around her. She spoke about how her daughters became more death literate through formal education, which helped them make sense of what was happening and prepared them for what lay ahead:

*Our youngest daughters*,* they went on a course about death*,* and you know. I remember one night*,* they were having their dinner*,* and they started moving beans around the plate and they said*,* ‘These are cancer cells and they’re not touching the rest of the body.’ But it was good because it gave them a very good understanding of what was going to happen.*

Others turned to different sources of support to navigate uncertainty. Kim found reassurance in the collective experience of online communities, *“The cancer of the oesophagus group who were amazing*,* I have to say*,* the Facebook support group.”* Meanwhile, Sarah’s professional background and familiarity with healthcare services enabled her to anticipate and plan for her father’s final days in a way that shaped her interpretation of his death, *“It was a beautiful passing […] no dramatic struggle or gasping for breath that you often see portrayed”*.

### Group experiential theme 4: Finding solace in connection

This theme reflects how participants found comfort and strength in connection with others as they adjusted to the reality of impending loss. Their accounts often conveyed a tension between remaining emotionally present and preparing both themselves and their loved ones for what was to come. Beth described this state as a *“living death*,*”* adding, *“It’s a living death*,* from diagnosis to death*,* and the people around are walking that living death with you.”* Abigail also reflected on the emotional weight of anticipated loss, recalling her husband’s grief for a future they would no longer share, *“He used to say*,* ‘all I wanted was ten years retired with you’*,* you know*,* that kind of thing. So*,* yeah… bitterness did creep in.”*

For some, this anticipation of loss brought an intensified awareness of time, of what remained, and how to make that time count. In response, many, like Beth, described turning to small, deliberate acts of connection as a way of sustaining closeness and creating meaning, *“I took her to the pantomime […] and we got her in the front row and that was just heaven with her sat with her blanket and her hat on.”* Rebecca reflected on how her and her mother, *“definitely spent more time together”.* In Jane’s case, organising an early birthday celebration became a way of honouring her mother’s wishes while adapting to the reality of her limited time:

*So, we had her 70th birthday party early because she’d always wanted a big party […]. But we knew she wasn’t going to make it […] So we arranged a big birthday party for the middle of July for her to have this lovely birthday.*

Deirdre echoed this focus on memory-making, sharing, *“we’d said to her “tell us things you want to do.” So*,* we managed to get away to a spa hotel and stuff like that*,* all the girls together.”* These acts, while practical, were underpinned by a desire to hold on to connection in the time that remained.

For Abigail, the phrase *“let’s cram in as much as we can”* conveyed both urgency and a yearning to find joy, and make the most of whatever time remained:

L*et’s go to the place that we love with our family. And then after that*,* that was kind of like the driving force because it was like*,* well*,* where should we go next and what should we*,* you know*,* it was like*,* let’s cram in as much as we can for as long as we can*.

Having these connections not only allowed participants to make meaningful use of the time they had but also appeared to help buffer distress and provide emotional strength. Rachel reflected on the bond she shared with her sister, *“Kathryn and I were closer than anything. So*,* you know*,* we held it together for each other*,* I think we found the strength to keep going*,* for each other.”* She also spoke to the significant presence of her wider support network, *“I have a very good network of friends too*,* and there was a lot of that support there that was going on.”*

### Group experiential theme 5: reframing perspective

This theme captures how caring for someone with advanced oesophago-gastric cancer often prompted a shift in how participants approached their lives going forward. For some, the experience of caring, and the loss that followed, served as a catalyst for re-evaluating their values and everyday priorities. In the aftermath of exhaustion and sacrifice, many described developing a clearer sense of what mattered most to them.

Some, like Abigail, reflected on how witnessing illness and death brought about a new attentiveness to time and mortality, *“I think it brings home your own mortality. You know*,* you’ve just got to live for the day”* For Beth, the experience of loss appeared to cement how she wanted to spend her life, *“This is my life. I’m only here for me and my kids […] it is precious*,* life is so very precious.”*

This attentiveness extended to a heightened appreciation for everyday moments. Sophie described how her perspective had changed, bringing both gratitude and longing, *“I appreciate everything now more. I appreciate what she did when I was younger*,* and I miss her so much. I miss her for the kids*,* and that I can’t talk to her about the little things.”*

Others reported behavioural shifts that reflected these changes in perspective. For Abigail, it led to a greater sense of spontaneity, *“It’s given me a bit of a devil may care kind of attitude really […] there’s nothing to lose.”* Sophie, too, described adopting a more selective approach to how she now chooses to spend her time, *“It has changed me in that*,* if I don’t want to do something. I don’t want to go somewhere or stuff*,* then I don’t do it.”* Sarah’s reflections suggested a more cautious but intentional orientation to time, shaped by her father’s premature death, *“It does change us […] when we think about something we want to do*,* but consider putting it off*,* well it adds an extra thought of ‘well actually*,* given what we know*,* is that still a good idea?”*

For Rachel, positively reframing her experience by focusing on what it had given her rather than what it had taken away enabled her to better cope with her loss:

*I’m trying my best to turn it around into being*,* well I don’t want to say positive*,* but it’s a privilege*,* to be with someone when their last breath is leaving them. You know that in itself is a huge privilege and I wouldn’t have had that in any other way.*

Sophie expressed a similar strategy, *“It was awful*,* but it was lovely at the same time? I felt I did have*,* there wasn’t a thing that we didn’t say or do*,* you know?”*

Whilst many of the participant’s narratives reflected positive reframing or growth, Jane’s reflections illustrated a different trajectory. Instead, her perspective was altered in ways that felt unsettling and unresolved, “*My outlook is completely different*,* I’m more of an anxious person now than I was before”* Unlike some of the other participants who found new clarity or meaning through their experiences, Jane described feeling emotionally unanchored following her mother’s death, *“I realise my mum was actually the anchor of the family and managed everything and when that anchor goes and they go*,* I think in the traumatic way that she did*,* I think that was the bit really*,* the fear*,* because we couldn’t make sense of what was happening.”*

## Discussion

This study is the first to explore the lived experiences of people who have provided informal care for individuals with advanced oesophago-gastric (OG) cancer. Using an interpretative phenomenological approach, the analysis sheds light on how participants made sense of and adjusted to a caring role situated within a rapid, uncertain, and often distressing illness trajectory. To support theoretical generalisability [55] and enhance interpretative insight, the discussion draws on psychological models of coping and adjustment to contextualise the findings.

Participants’ narratives highlighted how caring in the context of oesophago-gastric cancer was shaped not only by the emotional and physical demands of caring for someone with advanced illness, but also by the distinct characteristics of the illness itself. Consistent with previous research focused on patient experiences [19, 20, 29, 30], the findings reflect advanced oesophago-gastric cancer as a condition that often presents with vague symptoms, progresses rapidly, and results in significant bodily deterioration. This study extends that work by showing how these illness characteristics are experienced and interpreted by carers, who must respond to them while also managing their own lives and responsibilities. For carers in this study, these physical changes were not just clinical challenges but emotional and relational experiences. Participants described how feeding, weight loss, and bodily care disrupted shared routines, challenged relational identities, and triggered early grief responses, including anticipatory grief [65]. Tasks such as feeding a parent or bathing a partner raised complex feelings around dignity, intimacy, and loss, echoing findings from other cancer contexts where bodily decline interferes with interpersonal dynamics [66].

In response to these physical, emotional and relational challenges, how the participants’ appraised the situation played a significant role in shaping their coping efforts. Interpreted through the Common-Sense Model of Self-Regulation [67], two interrelated illness representations were particularly salient: control beliefs and coherence beliefs. Control beliefs refer to an individual’s perceived ability to influence the course of illness or manage related demands, while coherence beliefs concern how understandable, structured, or internally consistent the illness appears to the individual [68].

In this study, control beliefs were frequently undermined. Participants often described the illness as a relentless and unmanageable process, characterised by rapid physical deterioration and intense care demands. Many carers reported feeling powerless to change the course of events or ease their relative’s suffering. This erosion of perceived control contributed not only to emotional strain but also to patterns of self-neglect, where participants prioritised the needs of the person they were caring for over their own health. These findings are consistent with evidence linking low control beliefs to poorer self-care behaviours [69].

Coherence beliefs were also notably compromised. Extending previous research highlighting that oesophago-gastric cancer is poorly understood by the public and often absent from mainstream discourse [30], many participants entered the caring role with little prior knowledge or sense of what to expect. Clinical communication was often experienced as fragmented, or insufficient, making it difficult to construct a coherent narrative about the illness or anticipate its trajectory. This supports the view that developing a sense of coherence, or comprehensibility, is closely tied to how information is conveyed in palliative care [70]. In the present study, limited illness coherence appeared to undermine carers’ confidence in managing care and contributed to heightened anxiety, uncertainty, and isolation. These findings resonate with the growing body of literature on death literacy, which emphasises the value of accessible, open conversations about dying and death as a means of reducing fear, supporting preparedness, and promoting adaptive coping [71].

Coping responses reflected the unpredictable and emotionally disruptive nature of the illness. Consistent with the Transactional Model of Stress and Coping [72], participants described drawing on a range of strategies depending on how manageable the situation appeared at the time. Emotion-focused responses such as avoidance, suppression, or distraction were often used during periods of acute stress, especially when the situation felt overwhelming. These responses helped to contain emotional overwhelm but as shown in wider caring literature, were frequently associated with exhaustion and emotional withdrawal over time [42]. Meanwhile, as participants sought more active ways to manage care, many turned to problem-focused strategies such as information-seeking, accessing support, and planning. These responses were generally seen as more adaptive, enabling carers to navigate uncertainty and meet practical demands. This aligns with literature linking problem-focused coping to more favourable psychological outcomes in caring contexts [42].

Meaning-focused coping, described as the use of beliefs, values, and existential reflection to sustain meaning when stressors are chronic or unresolvable [73], was also evident within the findings. Participants described efforts to protect moments of connection, find purpose in their caring role, and reframe their experiences in ways that aligned with their core values. These responses helped sustain a sense of agency amid powerlessness and reflected an ongoing process of mourning and meaning-making. While meaning-focused coping is well-documented in the context of advanced illness and bereavement [74], this study highlights how the distinct features of advanced oesophago-gastric cancer, particularly the suddenness of diagnosis and rapid loss of normality, intensified the need for such coping. Notably, these meaning-making efforts appeared to support not only the endurance of distress but also moments of positive affect, such as gratitude or closeness, consistent with research suggesting that meaning-focused coping enables the coexistence of positive and negative emotions during ongoing stress [73].

For some, the period following bereavement prompted further reflection of meaning, with participants describing shifts in perspective that echoed aspects of post-traumatic growth [75]. While much of the literature conceptualises post-traumatic growth as an outcome that follows bereavement [76], the experiences described here suggest something more provisional. These changes were not framed as resolution or closure, but as a process in which participants sought to reinterpret their experience, find benefit, and redefine their values to support ongoing coping in the aftermath of loss [77]. This is consistent with work highlighting meaning-making as a central process in adjusting to loss [78, 79].

Taken together, these findings reflect the conceptual view of adjustment as a dynamic and context-sensitive process [47]. Coping responses did not represent fixed styles but fluctuated in line with perceived demands and available resources. Participants who were able to shift their coping strategies, tolerate uncertainty, reframe their circumstances, and find meaning in their caring role often appeared better positioned to sustain their own well-being. This fluidity aligns with the concept of psychological flexibility, the capacity to remain open and engaged in values-based action, even in the presence of distress [80].

### Clinical implications

While specific to this sample, the accounts highlight issues that may resonate more broadly across palliative and oncology care. Participants described how the illness often disrupted daily routines and relationships, pointing to the relevance of family-centred approaches that acknowledge the role of carers in advanced oesophago-gastric cancer care. Research shows that involving carers in discussions and care planning may help validate their role and ease relational tensions [81]. At the same time, the emotional and physical demands of caring meant many participants deprioritised their own needs. Services that explicitly support carer wellbeing, such as respite options, stress management, or peer-based support, may help to reduce longer-term strain and support more sustainable care provision. Participants also described a lack of information and fragmented communication, which contributed to feelings of uncertainty and isolation. Tailored education about disease progression, consistent clinical communication, and condition-specific guidance could help carers feel better prepared. In light of the rapid and often unpredictable trajectory of advanced oesophago-gastric cancer, earlier access to palliative care services may also support families in navigating uncertainty and anticipatory grief.

Finally, some participants found meaning in their caring role or described a shift in perspective following bereavement. While not universal, these accounts reflect the potential value of psychological support that makes space for meaning-making. Approaches such as Acceptance and Commitment Therapy [80] or meaning-centred interventions [82] may be helpful in supporting adjustment in this context.

### Research implications

From a research perspective, the findings offer in-depth insight into the adjustment challenges faced by carers of individuals with advanced oesophago-gastric cancer. They highlight the importance of both informational and emotional support, suggesting that psychoeducational components could form a valuable part of future intervention development [83]. Such approaches may help equip carers with clearer knowledge of the disease and its progression, while also supporting the emotional and psychological dimensions of their role. Future research could explore the design and feasibility of such interventions, ensuring that content meaningfully reflects the specific challenges described in this context. Timing and delivery methods should also be examined, particularly given the rapid and unpredictable nature of the illness trajectory.

The findings also extend earlier work with carers of oesophageal cancer survivors’ survivors [23], suggesting that carers’ illness beliefs may shape how they cope and adjust. Interventions that engage with carers’ personal understandings of the illness could be a promising avenue for improving psychological well-being, though further work is needed to explore this in depth.

Finally, given that adjustment was experienced as a dynamic process shaped by changing demands, beliefs, and coping responses [47], longitudinal research is needed to trace how carers’ experiences and support needs evolve over time. Such work could help inform the development and timing of tailored interventions that respond flexibly to carers’ trajectories of adjustment.

### Public health implications

Participants’ accounts indicated that oesophago-gastric cancer was often experienced as a “*lesser known*” condition, with its complex symptoms and rapid progression poorly understood both publicly and professionally. This perceived lack of awareness appeared to intensify feelings of uncertainty, isolation, and unpreparedness, suggesting a need for broader societal understanding of the disease, its symptoms, and likely trajectory. Targeted public health initiatives could help raise awareness and support earlier recognition of symptoms.

Incorporating the concept of death literacy [71] into public health and education strategies may also be valuable, particularly for cancers with poor prognoses. Increasing public understanding of end-of-life processes, including palliative care, advance care planning, and informal caring, could help normalise these experiences, potentially reducing stigma and facilitating better preparedness and support for carers.

More broadly, these findings align with the principles of compassionate communities [84], which advocate for the shared responsibility of care across health systems and communities. Strengthening informal networks and community-based resources may help buffer the emotional and practical demands placed on informal carers and indirectly enhance the quality of care received by patients.

### Strengths and limitations

This study offers several important strengths, while also presenting limitations that should be acknowledged. It is the first known exploration of the adjustment experiences of bereaved informal carers of individuals with advanced oesophago-gastric cancer, addressing a significant gap in the psycho-oncology and caring literature. The study contributes novel insights into the emotional, existential, and relational dimensions of caring and bereavement in this context. In line with best practices for high-quality IPA [64], the analysis draws on participants’ own words to construct a nuanced interpretative account, capturing both shared and idiographic aspects of sense-making. The retrospective nature of the interviews allowed for reflection across the illness and bereavement trajectory, enabling temporal depth that is often difficult to capture in cross-sectional studies. However, these retrospective accounts were shaped by memory, which is known to be influenced by both time and emotion [85, 86]. The variability in time since bereavement may have introduced inconsistency in recall, with the potential for distortion or selective remembering [85, 86]. Although this reflective stance is valued in IPA, it limits the capacity to assess change over time in a systematic way.

The sample was small and homogeneous, consistent with IPA’s idiographic focus [55], but all participants identified as white and female. This limits the transferability of the findings to carers from other ethnic, cultural, gendered, or socio-economic backgrounds. Future studies should seek to include a more diverse range of carer relationships and backgrounds.

Interviews were conducted remotely via video conferencing or telephone, which may have shaped the dynamics of data collection. While this may have constrained rapport-building, it also allowed participants to speak from familiar settings, likely enhancing comfort and enabling broader geographic inclusion during a period of public health restrictions.

The researcher’s own experience of caring for her mother with advanced oesophago-gastric cancer, and later navigating her death, is a key reflexive consideration. This commonality may have helped support trust and emotional safety during interviews. At the same time, it may have influenced the dynamics of disclosure, with participants potentially emphasising experiences they felt the researcher would understand. Rather than being viewed as a limitation, this is recognised as part of the interpretative process that IPA explicitly embraces [55, 60].

The small sample size may be viewed as a limitation in terms of the study’s statistical generalisability. However, this reflects the methodological commitments of IPA, which prioritises depth of engagement with individual experience over breadth [55]. Rather than aiming to produce generalisable claims about all carers of people with oesophago-gastric cancer, the study offers theoretical generalisability [55]. In this way, the findings provide both a detailed, idiographic account of adjustment and a conceptual contribution that may inform future research and practice across comparable palliative and caring settings.

## Conclusions

This study, using an interpretative phenomenological approach, offers a nuanced account of the lived experiences of bereaved carers of individuals with advanced oesophago-gastric cancer. It highlights the complex and shifting emotional, relational, and practical demands they faced within a care context characterised by urgency, uncertainty, and limited preparation. Coping responses evolved in relation to changing circumstances, personal beliefs, and available resources. Strategies such as emotional containment, problem-solving, and meaning-making were drawn on as they negotiated the realities of care and loss. These responses shaped how they made sense of their role and supported their capacity to adapt, both during the illness and beyond bereavement. Overall, the findings suggest that adjustment in the context of advanced oesophago-gastric cancer care is an ongoing, context-sensitive process that continues long after the death of the person receiving care. While not intended to be generalised, these findings offer conceptually rich insights into this under-recognised caring context, with potential to inform both practice and future research.

## Electronic supplementary material

Below is the link to the electronic supplementary material.

Supplementary Material 1

## Data Availability

The datasets generated and/or analysed during the current study are not publicly available due to the sensitive and identifiable nature of the qualitative data, but anonymised excerpts are available from the corresponding author on reasonable request.

## Abbreviations

CSM: Common sense model
HCP: Healthcare professional
IPA: Interpretative phenomenological analysis
NICE: National institute for health and care excellence
UK: United kingdom
