# Supplementary material for: “You’re just in such crisis mode…frantic to get through the next day”: an interpretative phenomenological analysis of adjustment experiences among carers of patients with advanced oesophago-gastric cancer
Source: BMC Palliat Care. 2025 Jul 1;24:171. doi: 10.1186/s12904-025-01810-6 (PMC12211477; doi:10.1186/s12904-025-01810-6)

Supplementary Material 1: Participant topic guide

Supplementary Material 2: Table of participant group experiential themes (GETs)

Supplementary Material 3: COREQ guidelines for the reporting of qualitative research (Tong, Sainsbury & Craig, 2007).

**Supplementary Material 1: Participant topic guide**

| **Main Topic** | **Topic guide** |
| --- | --- |
| **Introduction** | - Introduce self to participant - Explain interview process - (timescale, confidentiality) - Recap study aims - Explain use of the participant’s information, confidentiality (including limits) and right to withdraw. - Undertake informed consent with participant (if not already completed) - Completion of socio-demographic form - Explain under what circumstances the researcher might stop the interview (e.g. signs of distress). - Provide the participant with the opportunity to ask any questions - Begin recording |
| **Background and general understanding of illness** | To begin the interview, we will ask a very general question to develop an understanding of the background to their close persons diagnosis:  Could you tell me about your experience of your close person being diagnosed with oesophago-gastric cancer?  **Prompts:**   - How did this make you feel? - Why did you feel this way? - How did you make sense of that? - How did you manage that? - How did your close person manage this? - How did you talk about this? - Did you know what to expect? - Did you feel adequately supported when given the news? |
| **Physical Impact and Challenges to Adjustment** | Could you tell me about the physical challenges faced by both yourself and your close person as a result of oesophago-gastric cancer?  **Prompts:**   - What specifically made you feel that way? - How did you manage this? - How did your close person manage this? - Did either of you avail of any services to help you manage? - Did you experience any practical issues? Ie. with work, childcare? - How did you maintain your own physical health? - Did this change over time? |
| **Psychological Impact and Challenges to Adjustment** | Could you tell me about the emotional challenges faced by both yourself and your close person as a result of oesophago-gastric cancer over time?  **Prompts:**   - What specifically made you feel that way? - How did you manage this? - How did your close person manage this? - Did you feel able to talk about your feelings and worries? - Did either of you avail of any services to help you manage? - Did this change over time? |
| **Social Impact and Challenges to Adjustment** | Could you tell me about the social challenges faced by both yourself and your close person as a result of oesophago-gastric cancer?  **Prompts:**   - What specifically made you feel that way? - How did you manage this? - How did your close person manage this? - Did either of you avail of any services to help you manage? - Did this change over time? |
| **Existential Impact and Challenges to Adjustment** | Could you tell me about the impact your close-persons diagnosis had on your outlook on life?  **Prompts:**   - What specifically made you feel that way? - How did you manage this? - How did your close person manage this? - Did either of you avail of any services to help you manage? - Did this change over time? |
| **Informal care and support** | Could you tell me about your role as a carer?  **Prompts**   - How did this make you feel? - What specifically made you feel that way? - How did this change your family life? - How did you manage this? - How did your close person manage this? - Did this change over time? |
| **Ending the interview** | Recap briefly on researcher’s understanding of what was said – check if there is anything they would like to elaborate on, or anything not discussed that they feel is important to mention   - E.g. would you like to share any other thoughts or feelings about oesophago-gastric cancer?   Discuss whether they felt involvement was worthwhile  Thank again for taking part  Remind participant that can have a summary of the project findings (dependent on providing an email address) |

# **Supplementary Material 2 – Table of participant group experiential themes (GETs)**

| **Group Experiential Theme 1: Losing Ground** | |
| --- | --- |
| Carers faced an overwhelming and disruptive experience as they navigated the unpredictable and severe effects of advanced oesophago-gastric cancer. Their lives were consumed by caring duties, leaving little room for personal autonomy or self-care. Access to supportive resources, like clinical nurse specialists, played a crucial role in helping carers cope amidst the relentless demands. | |
| **Abigail** | *“You are in a whirlwind because your whole life becomes a round of appointments and procedures, and that kind of carries you along.”*  *“You feel like you’re on this conveyor belt, and they’re saying do this, do that.”*  *“I found there was not much time in between to think of anything else and you know, of myself, because, it was just he was the priority.”*  *“I had a brilliant specialist Nurse who was my lifeline. And I could ring her, and she would get an appointment for me or anything like that.”* |
| **Beth** | *“I cried in bed because of the situation, but then I got up in the morning and got on with it. You don't I don't know how you do it, because it’s all consuming, but it's not. You’re living a life that’s not real. I mean, it's just you’re in a bubble.”* |
| **Deirdre** | *“I didn’t seek out specific support really, because I didn’t really have time to do any specific support.”*    *“We were having to make big sacrifices to be able to care. You know it was very difficult for us to do that. And we didn’t get any support.”*  *“I actually feel that we had six months of Mummy, from diagnosis to death, of not being recognised. We were like we were basically her only support.”* |
| **Jane** | *“It was like a roller coaster speeding out of control, yeah, it was really bad.”*  *“So by the end of it, I’d also lost a stone and a half in weight and kind of had become anorexic without realising it. So about two months after Mum died, I went back to work and collapsed at work and ended up in hospital. And they tried to give me a blood transfusion, emergency, blood transfusion, and they couldn’t do it because all my veins had collapsed. So consultants came down saying, you know, you look, your profile is as if you’ve been a drug, but there’s nothing in you. And I was like, no. So we can only think you’ve been in a major disaster, have you been in a war?. Well, I was thinking I have been in a major disaster for me, but probably not for you.”*  *“I feel like she wasn't treated like a person, that she was a number that just went through on a conveyor belt. There was no empathy for her or for my dad.”* |
| **Kim** | *“I was 3 hours away, I had a daughter at uni, and the dogs, and you know, it was hard […] it was difficult. I had a life... So, for me to work, and then drive down on a Friday and back on Sunday[…] or to come back in one day[..]. But it had to be done.”*  *“I’d stand in the garden, have the odd tear, then pull myself together.”* |
| **Maureen** | *“At the time you’re just in such crisis mode. You’re just frantic to get through the next day.*    *“Everything just stopped and I didn’t even realise everything had stopped until a few months after dad had gone, and I got into a good conversation with a girl, and I said, I’ve just decided that it’s time for life to start. That’s when I realised that I had just stopped the clock. I think I kind of knew that it was the last year I was going to have with dad, so I just stopped the clock. So, yes, everything just stopped.”*  *“It all happened so fast and at the end, everything just seemed to happen within sort of fortnight.”* |
| **Rachel** | *It was very very tough. I don’t even just mean emotionally, I mean physically and emotionally, every sort of emotion you’re going through.”* |
| **Rebecca** | *“I think at the time I was just in this sort of in this. ‘Keep calm, carry on’ stage, but I can’t remember. I just kinda did what I had to do, kind of thing.”*  *“I think when you go through something like that you don’t realise just how affected you are. So, I didn’t at the time, I didn’t seek any support from Macmillan or anything like that, not at all, and neither did my brother.”*  *“My friends would tell me I was drinking too much.. a bit too far, but they understood it.”*  *“I lived with a friend who had a terminally ill parent so when I was at the house, it was nice to talk to them sort of thing.”* |
| **Sarah** | *“Essentially, I feel like we were left to it […] I don’t believe there was anything offered. No, I think it was a done deal. They knew he was going to die; they knew he didn’t have long. Not ‘what was the point,’ but you know, just as if he was already dead in their eyes, that’s how I feel.”*  *“I wonder if that is because I presented as a capable, physically and emotionally capable person with some medical knowledge? Like, had I been falling apart at the seams, and incapable of physically supporting and dealing with my dad, I wonder if something else would have been there? But I never once said ‘no I don’t want that, I can manage’ I just got on with it. I don’t believe there was anything offered.”* |
| **Sophie** | *“And I think you're just, at the time, you're just twenty four hours a day and I'm frankly thinking […] nothing's too much difficulty and I would literally have stirred her tea if I thought that would help.”*  *“I went to every appointment and tried to think positive, but looking back, no, I was just exhausted, but I just did it cause I didn’t want to not do it, you know.”*  *“I remember sitting in the bathroom having to tidy up what had gone wrong. And I just started crying, saying ‘I can’t do this anymore. I physically cannot do this anymore.’ And she couldn’t do it anymore.”*  *“And it was like once you’ve stepped on the conveyor belt, you have to go to the end. And I felt that there should be options along the way to say “No”* |
| **Group Experiential Theme 2: Shifting dynamics** | |
| Carers struggled with the rapid decline of their loved one’s abilities due to advanced oesophago-gastric cancer. Tasks like feeding or preparing meals were emotionally distressing, challenging traditional roles within families and requiring carers to navigate new relational dynamics. | |
| **Abigail** | *“I remember thinking, like, I’ve become a nurse and a carer more than a wife. You know, it was all very clinical.”*  *“With him being sick so often… there was risk of him getting dehydrated, so I was having to squirt water through the feeding tube, you know, to keep him hydrated and I’d say that was when, he was at his best, as well as he was, you know. Its only now that I’m talking about it that I realise, how bad it was really.”*  *“I knew physically, you know, it was like, you know, taking over him. You know, for someone to go through all that, but I’d kiss his lips and he couldn’t even feel it because his lips had gone numb because he had a massive tumour under his chin.”*  *“And if he felt I was getting kind of too sympathetic or whatever? He would, well when I say annoyed, he wouldn’t shout at me, but it's sort of like, you know, I'm still me, don't treat me like light, because that's not how you normally treat me.”*  *“He used to hate, you know, towards the end he needed me to help him get into the bath. He just didn't have the strength to get himself in and out of the bath and we hated that. You know, he used to say, look at the state of me, let me get some clothes on quickly, you know,. He just hated what it was doing to him.”* |
| **Beth** | *“It’s very hard to watch, incredibly difficult to watch. Seeing my mum eat baby food, it was horrific (...) You know, for a grown adult, it’s so demeaning, not only are they ill, but then they’re treated like a child.”*  *“Every day I would make her lunch and she would say, this isn’t what it was supposed to be like. When I would be trying to feed her a spoonful of soup or ice-ream. And you know, heart breaking as it was, you just did it because you had to. You know, I had a family, I had 3 kids.”* |
| **Kim** | *“He was the best host going. He always made sure everybody had a good time […] But towards the end, once he really couldn’t swallow. So a lot of that stopped then. He was struggling for a little while, so there were no Friday nights down the pub or anything.”* |
| **Maureen** | *“I think it is such an anti-social thing, and the voice too. He was a real character, like he was the narrator of so many plays, and any cancer robs people of so much, but this cancer robbed him of his lovely voice and that was a huge loss”.*  *“That’s big for oesophageal cancer, because other cancers that isn't something that happens so that's definitely a unique aspect of it. And it’s also that antisocial thing of being sick.”*  *“It was difficult because there was a battle of wills between mum and him, because mum wanted to wrap him up in cotton wool and make sure nothing touched him. To protect him. Whereas for dad, he might as well have been dead. That wasn’t life to him. He wasn’t able to do the things that he wanted to do. He just wanted to run to the finish line but mum was constantly pulling in the other direction.”*  *“I was just like, right, this is what we're doing, and we need to get through this, and we need to do it together.”* |
| **Rachel** | *“Oesophageal cancer, it just takes your basic ability to eat and drink away from you, so if you imagine, on the hottest day of your life, and you cannot get a drink. Just imagine how horrible that feeling is.”*  *“It was really hard to watch someone like her, who was very vivacious, and the life and soul of the party, you know. She was Mrs Christmas, and she loved her food, loved cooking for people all that kind of stuff. It became, hard, to watch someone who loved their food struggle to enjoy the things that they used to enjoy.”*  *“She would have hated that she was upsetting me, and I know that’s how she would have been thinking, but I wouldn’t have been anywhere else in the world, you know. Nowhere. I was going to be there.”*  *“Just seeing her like that, it was just devastating, but I wouldn’t have had it any other way, you know.”* |
| **Rebecca** | *“Yeah, I think we were all treading on eggshells a little because we didn’t want to be that person who, you know, said the wrong thing, or upset her, you know, so yeah, I think I did have to change a bit”*  *“Yeah, it was just getting everything that she needed really.”* |
| **Sarah** | *“I went to look after him, but I was shocked by his appearance. He looked jaundice to me, he’d lost weight, he just wasn’t my dad.”*  *“I think it was at the point that I arrived that the relationship between my mum and I shifted, between her being my mum, and me becoming and adopting the role of a carer. The relationship shifted. I was the one then that sorted things out, dealing with things. My mum was the one that deferred to me. It was like the roles had reversed.”* |
| **Sophie** | *“She didn’t have the strength to do it. Cause her hands couldn’t deal with turning those knobs, putting things together. She didn’t have the ability to do it herself. So, she did depend on somebody else to do it.”*  *“It’s human nature to feed everybody and support them. You love somebody by feeding them, giving them food, and making sure they’re all right. And when you can’t socialise, can’t do that, it’s very difficult.”*  *“You just don’t think you should be doing certain things for your mother. You shouldn’t have to do that.”* |
| **Group Experiential Theme 3. Navigating the unknown** | |
| Many carers found themselves thrust into a world of unfamiliar medical terminology and insufficient knowledge about managing advanced oesophago-gastric cancer and the path ahead. Seeking information and understanding became essential coping strategies, empowering carers to navigate the complexities of the disease and its implications effectively. | |
| **Abigail** | *“I remember going into that appointment, because we knew it had come back and he knew from how he felt that it must have been serious because this is where he started getting numbness and things. And he just said “if she gives me 2 years, thatll be good. “ So I think he went in thinking he was going to be told something like that, so when she said six months, his face was…I think it was the first time I saw it, like as if he was the beaten, kind of like, like bloody hell, this is it. So I was angry at how she dealt with that.”* |
| **Deirdre** | *“One doctor (…)was brilliant… he was great and he explained everything very, very well.”* |
| **Beth** | *“I had never heard of the oesophageal cancer. Never had heard of it and I mean, now I’m like the oracle on it. Cancers one thing, but this type of cancer, unfortunately, you do become like Google, you know everything. You know, you get hyper vigilant. And I think that will stay with me forever. You never get over it. It’s something that’s there in you… it’s just there.”*  *“I came home and I was on it.. completely. But I would be very much a researcher to find out all that we’d need to know, all the information.”*  *“Our youngest daughters, they went on a course about death and you know. I remember one night, they were having their dinner and they started moving beans around the plate and they said ‘These are cancer cells and they’re not touching the rest of the body.’ But it was good because it gave them a very good understanding of what was going to happen.”*  *“I would love, love there to be something that would make it easier for people to have like, OK, so breast cancer and cervical cancer and all those things people can go and avail themselves of, whether they up take that or not. That’s not the issue. It’s there. It’s there. But ultimately, ultimately, I would like that to be the situation for this cancer. And there has to be risk groups and establishing those groups, and I personally would do whatever it takes to establish them to help that situation along.”* |
| **Jane** | *“I didn’t feel like I was doing a good enough job […] it was really difficult, and we weren’t sure if Mum was in pain because she would groan every time.”*  *“She just screamed and screamed and screamed. And it was just, she was so distressed, and she didn’t know who any of us were… So they just didn’t tell me what would happen when she got completely sedated. So we didn’t get to say goodbye.”*  *“And so basically, I think we just went away from there thinking that actually they were all really rubbish. And it’s really sad. They were rubbish and we’re really sad for mum that they were rubbish, but that was it.”* |
| **Kim** | *“In the hospital was very hard, and you know, trying to get answers from the consultant was very hard”*  *“Mum and dad wanted answers in regard to timings, but they couldn’t get them. […] But I think at that point, people want answers and to know how long they have got left. But we couldn’t get the answer.*  *“Even if dad hadn’t asked, I think dad wa.”nted to know what next stage was, what the next steps were. Cause he just felt so ill. So, he just needed to know what to expect for the next step, but we never got that.”*  *“And that’s, that’s the thing, on the cancer of the oesophagus group, who were amazing, I have to say, the Facebook support group. Theres a woman there, and we message each other occasionally, because she was there when my dad, you know, and her dad.”*  *“I think the key thing is there has to be more awareness out there. There has to be some kind of campaign, people need to be aware that if you’re on Rennie’s or Zantac, or something, by that point, that needs to be an alarm bell for you. Its fine popping a few Rennie’s, but when you get to needing them every day, then it’s a problem”.* |
| **Maureen** | *“It’s the joined-upedness for want of a better word….between the various departments, between the hospital and the GP, the district nurses, all the charities. I think that’s a common thing, whether it’s medicine or education but no matter what it is, everyone seems to be operating in their own bubble. You know, instead of that overarching approach that would benefit people.”*  *“It was more relaxed, whereas the hospital, it was a hospital, this was much more a hospice environment. So it was great for us as a little bit halfway house. It was it was relaxed. We could come and go any time of the day or night. It definitely was easier.*  *“I don’t want to start saying “my cancer is worse than your cancer” . But there are some more hidden ones than others in the world.”*  *“There were so many people involved. You were going “do I ring the district nurse.” “Do I ring the GP” “do I ring the pharmacist at the hospital?”*  *“You know, in that whole year the GP called at the house once. It was horrendous, absolutely horrendous, and things got so bad.”* |
| **Rachel** | *“There was a long delay from having Barrett’s to oesophageal cancer. And by the time the surgeon got to her when she went through her surgery in July. He said this should have been picked up much much much sooner.”*  *“I am someone that needs to know the information, and what we’re dealing with. So, I flew over with her, and we obviously, her and her husband and I went to the surgeon. I needed to know what this was.”*  *“I could not have faulted what her team did. We went through a terrible time with her, when she came home. […] So, for him to do that or someone who is already terminal, tip my hat to him, I really do.”* |
| **Rebecca** | *“When she came back from appointments, she would be upset, but then she would stop. And then she would be googling online and that kind of thing. But even up until the very end, I think she knew it was bad, but she still thought it would be okay, you know, even though she was stage four.”*  *“Until she was diagnosed, we didn’t know you could get cancer there.”* |
| **Sarah** | *“I don’t understand why oesophageal cancer is not on people’s radar.”*  *“I mean he was a classic oesophageal cancer patient. And I had to make the connection, and I am absolutely livid about that to this day.”*  *“It was a beautiful passing….there was no […] dramatic struggle or gasping for breath that you often see portrayed.”*  *“I shared details on Facebook of the symptoms and there was a lady in the village that we live in, who recognised the symptoms in her mum and got her to the doctor, and she credits me with being the person that opened her eyes to what the symptoms meant.”* |
| **Sophie** | *“wanted to know, like “well, what does this do or what does it feel like after this?” You're having chemotherapy and radiotherapy, but as I say, the clot, the pneumonia, everything. Nobody told her how she would feel. Nobody actually spoke, to say, “this is how it’s going to be, to go in your body”. You need somebody to tell you the truth, not just the medical bits..”*  *“I was always there when they talked to mum, and the doctors were very good when talking to mum. I do think that the cancer centre was good. They were on the ball about what they were doing, they explained, they were very understanding. They did everything they could.”*  *“I felt like there’s got to be somewhere that you could set up a system for the next person that needs help. Like a volunteer team that knew how to help, and they’ll support you for the first fortnight. And be at the end of the phone, if you call and say, “This beeper is going, what have I done wrong?”* |
| **Group Experiential Theme 4. Finding solace in connection** | |
| Anticipatory grief was a prevalent experience among carers, who struggled with the imminent loss of their loved ones. Amongst this, many found solace in cherishing present moments and creating lasting memories, highlighting the capacity for finding meaning in the face of advanced illness. | |
| **Abigail** | *“You know, let's go to the place that we love with our family. And then after that, that was kind of like the driving force because it was like, well, where should we go next and what should we, you know, it was like, let's cram in as much as we can for as long as we can.”*  *“He used to say, ‘all I wanted was ten years retired with you’, you know, that kind of thing. So, yeah… bitterness did creep in.”* |
| **Beth** | *“It’s a living death, from diagnosis to death and the people around it are walking that living death with you. So you start grieving from the day they’re diagnosed, but you don’t know that, and that’s the scary bit.”*  *“He had done everything, it had all been planned. So everything was done with military precision, military precision. So there wasn’t all that to do. […] So all we had to do was turn up, he didn’t want his wife or anybody to have to do that for him. He wanted it done, he didn’t want them to worry about it. Then, despite him hurting, he was always so thoughtful. There was a gift for me and his daughter. It was a necklace, a heart, and I have his fingerprint on the back of it.”*  *“So, all five of us were up at my mum’s and she told us. And she said, you can cry now, but I don’t want to see you cry again after this.”*  *“I took her to the pantomime […] and we got her in the front row and that was just heaven with her sat with her blanket and her hat on.”* |
| **Maureen** | *“*I*t was all about making memories.”* |
| **Deirdre** | *“She had like a bucket list that she had, now this was before the surgery was cancelled, and we’d said to her “tell us things you want to do.” So we managed to get away to a spa hotel and stuff like that, all the girls together.”* |
| **Jane** | *“So we had her 70th birthday party early because she’d always wanted a big party […]. But we knew she wasn’t going to make it […]So we arranged a big birthday party for the middle of July for her to have this lovely birthday.”* |
| **Rachel** | *“Kathryn and I were closer than anything. So you know, we held it together for each other, I think we found the strength to keep going, for each other.”*  *“I’m trying my best to turn it around into being, well I don’t want to say positive, but it’s a privilege, to be with someone when their last breath is leaving them. You know, that in itself is a huge privilege, and I wouldn’t have had that in any other way.”*  *“I have a very good network of friends too, and there was a lot of that support there that was going on.”*  *“When you’re going through something like that, you need to just not be part of the negativity, you need positively around you.”* |
| **Rebecca** | *“We definitely spent more time together, actually.”*  *“Me and my brother actually slept in the hospital, in a family room and that kind of thing.”* |
| **Sarah** | *“There was a really good friend of my dad’s, and he came and was so lovely because obviously my dad wasn’t eating and drinking much at the time. A friend of mine worked in a hospice and said why don’t you try sorbet. I remember his friend had gone all over to get hold of sorbet and brought it and my dad really enjoyed it because it was cooling and obviously it went to nothing so could go down.”* |
| **Group Experiential Theme 5. Reframing perspectives** | |
| The caring experience often prompted a revaluation of life priorities and values among carers. Some even adopted a more proactive and appreciative approach to life following the deaths of their loved ones. | |
| **Abigail** | *“I think it brings home your own mortality. You know, you’ve just got to live for the day.”*  *“It’s given me a bit of a (…) devil may care kind of attitude really. You know, whereas I might have been a bit like “oooh is that appropriate” whereas, you know, I don’t care now. I don’t mean that in a rude, or doing something wrong way, but ore in a “go get it, nothing in the way” like there’s nothing to lose”* |
| **Beth** | *“This is my life. I’m only here for me and my kids […] it is precious, life is so very precious.”* |
| **Deirdre** | *“I can try my best to eat healthily. I exercise, I do all things that I've been told I have to.”* |
| **Jane** | *“My outlook is completely different, so I’m more of an anxious person now than I was before.”*  *“I realise my mum was actually the anchor of the family and managed everything and when that anchor goes and they go, I think in the traumatic way that she did, I think that was the bit really, the fear, because we couldn’t make sense of what was happening and we couldn’t get any help.”* |
| **Kim** | *“I’m aware of how short life is, and how rapidly it can go wrong. I'm very grateful to still be here.”*  *“There’s a part of me, that knows life is very short. And what's around the corner? I'd like to get my work life balance under control. I spend every week trying to get my work under control. So that I can actually have some more “me time”* |
| **Rachel** | *“I’m trying my best to turn it around into being, well I don’t want to say positive, but it’s a privilege, to be with someone when their last breath is leaving them. You know that in itself is a huge privilege and I wouldn’t have had that in any other way”.*  *“I just try to think about the happier times, but I just wish we had longer with them. But I don’t want anybody to go through that, so if there was no cure for her, you wouldn’t want them hanging around with that pressure on them, because its just too much for their bodies, you know.”* |
| **Sarah** | *“It does change us, so much as we, aren’t so much as doing as we please whenever we please, but when we think about something we want to do, but consider putting it off, well it adds an extra thought of ‘well actually, given what we know, is that still a good idea?’”* |
| **Sophie** | *“It has changed me in that, if I don’t want to do something. I don’t want to go somewhere or stuff, then I don’t do it.”*  *“I don’t think you appreciate your parents until you’ve not got them.*”  *“I appreciate everything now more. I appreciate what she did when I was younger, and I miss her so much. I miss her for the kids, and that I can’t talk to her about the little things.”*  *“It was awful, but it was lovely at the same time? I felt I did have... there wasn’t a thing that we didn’t say or do, you know?”* |

# **Supplementary Material 3: COREQ guidelines for the reporting of qualitative research (Tong, Sainsbury & Craig, 2007).**


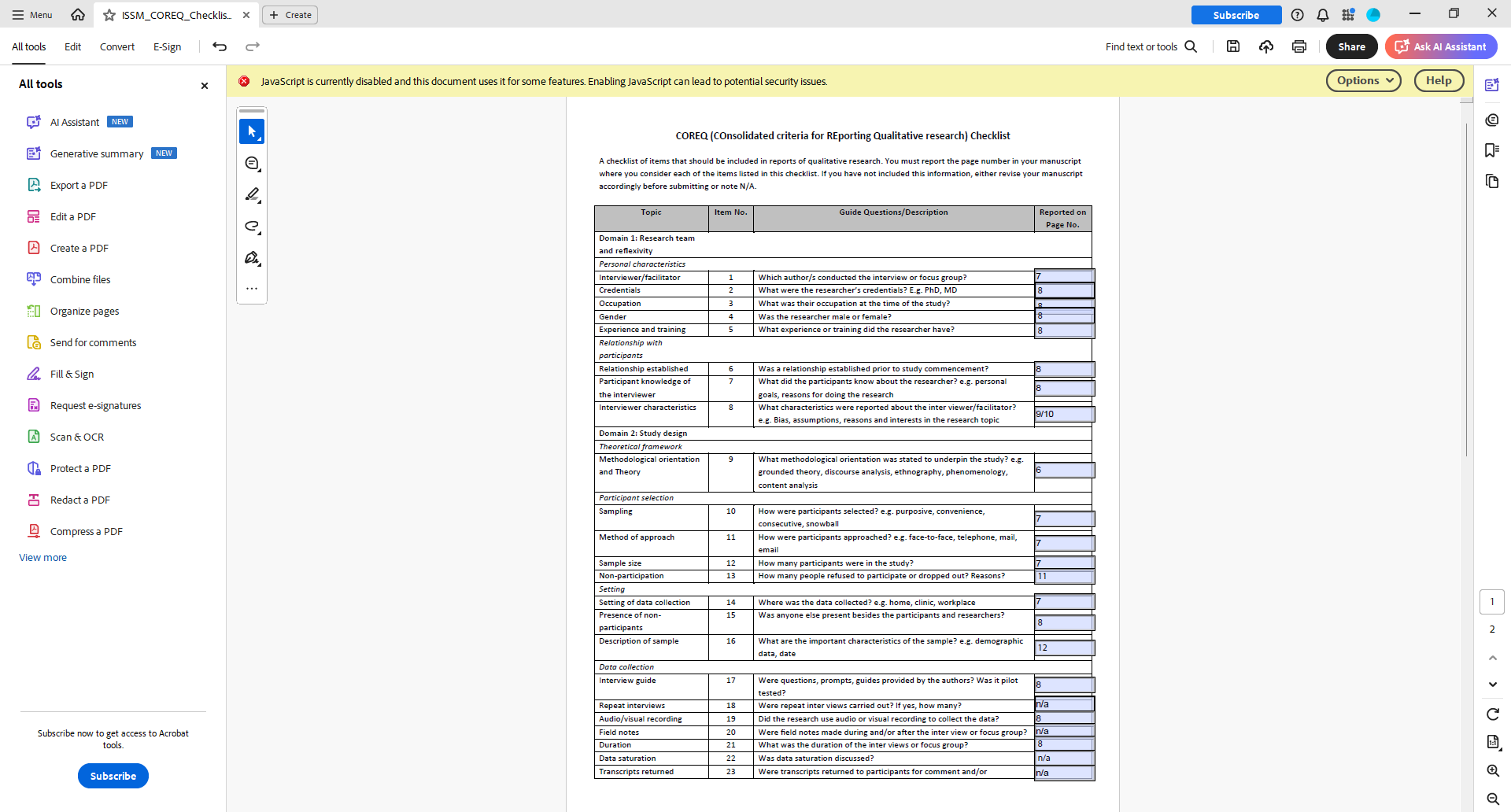


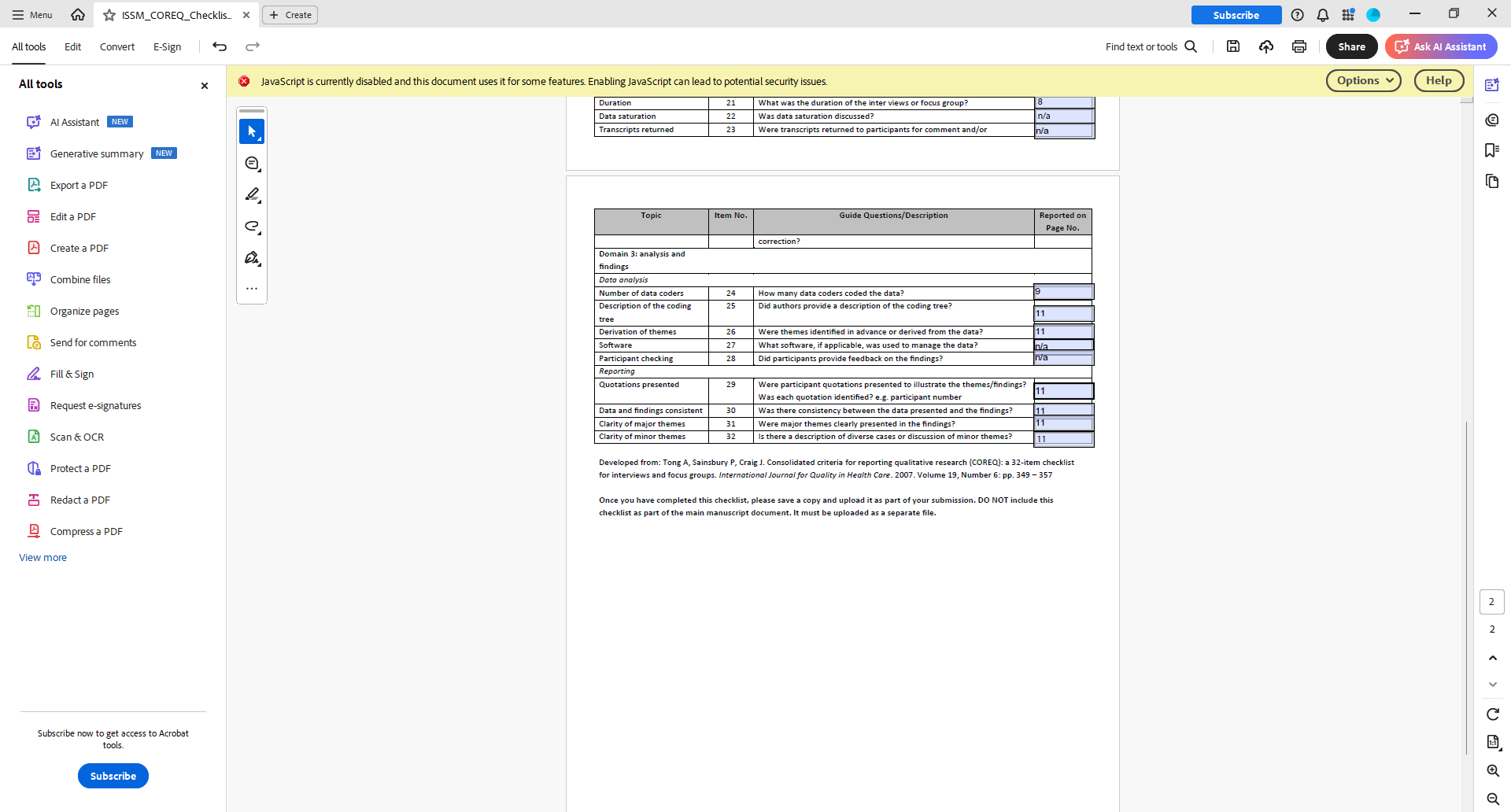

Supplement: Supplementary file 1 — Supplementary Material 1 [file 12904_2025_1810_MOESM1_ESM.docx]
